# Parental age and gene expression profiles in individual human blastocysts

**DOI:** 10.1038/s41598-018-20614-8

**Published:** 2018-02-05

**Authors:** Kiyotaka Kawai, Tatsuya Harada, Tomonori Ishikawa, Rikikazu Sugiyama, Toshihiro Kawamura, Atsumi Yoshida, Osamu Tsutsumi, Fumitoshi Ishino, Toshiro Kubota, Takashi Kohda

**Affiliations:** 1 Department of Epigenetics, Medical Research Institute, Tokyo Medical and Dental University (TMDU), Tokyo, Japan; 2 Department of Comprehensive Reproductive Medicine, Tokyo Medical and Dental University (TMDU), Tokyo, Japan; 3 AMED (Japan Agency for Medical Research and Development), Tokyo, Japan; 4 Department of Reproductive Medicine, Kameda Medical Center, Chiba, Japan; 5 Department of Reproductive Medicine, Kameda IVF Clinic Makuhari, Chiba, Japan; 6 Division of Reproductive Medicine, Sugiyama Clinic, Tokyo, Japan; 7 Denentoshi Ladies Clinic, Reproductive Center, Kanagawa, Japan; 8 Reproduction Center, Kiba Park Clinic, Tokyo, Japan; 9 Sanno Hospital, Center for Human Reproduction and Gynecologic Endoscopy, Tokyo, Japan; 10 Tokyo Kyosai Hospital, Tokyo, Japan

## Abstract

The epigenetic status of the genome changes dynamically from fertilization to implantation. In addition, the physiological environment during the process of gametogenesis, including parental age, may affect the epigenome of the embryo after fertilization. It is important to clarify the influence of parental age on gene expression in the embryo in terms of transgenerational epigenetics to improve the techniques currently used in assisted reproductive medicine. Here, we performed single-embryo RNA-seq analysis on human blastocysts fertilized by intracytoplasmic sperm injection, including from relatively elderly mothers, to elucidate the effects of parental age on the embryonic gene expression profile. We identified a number of genes in which the expression levels were decreased with increasing maternal age. Among these genes, several are considered to be important for meiotic chromosomal segregation, such as *PTTG1*, *AURKC*, *SMC1B* and *MEIKIN*. Furthermore, the expression levels of certain genes critical for autophagy and embryonic growth, specifically *GABARAPL1* and *GABARAPL3*, were negatively correlated with advanced paternal age. In addition, levels of transcripts derived from major satellite repeats also decreased as the maternal age increased. These results suggest that epigenetic modifications of the oocyte genome may change with parental age and be transmitted to the next generation.

## Introduction

The regulation of gene expression in the period between gametogenesis and embryonic implantation is closely controlled; it is both biologically and medically important because it occurs in the first stages of individual development. However, due to the small number of cells present, comprehensive gene expression analysis has only begun in recent years^1,2^. Various parental and environmental factors influence the transcriptome and epigenome of the embryo; as a result, the successful development and the delivery rate of embryos may be influenced. Among these factors, one of the most important challenges facing human *in vitro* fertilization (IVF) is the decline in pregnancy rate observed with advanced maternal age. It is believed that the major cause of this reduced pregnancy rate is aneuploidy caused by chromosomal nondisjunction during oocyte meiosis. The relationship between embryonic chromosomal aneuploidy and maternal age has been well-characterized, and significant evidence has been reported. Since pre-implantation genetic screening (PGS) was first proposed in 1993^3^, clinical PGS via trophectoderm (TE) biopsy has been widely utilized, and large-scale clinical studies have been performed to investigate TE biopsies. For example, Franasiak *et al*. reported human embryo aneuploidy based on TE biopsy^4^. McCoy *et al*. analysed day 3-blastomeres and TE biopsy samples using a single nucleotide polymorphism (SNP) array to characterize the timing of aneuploidy in relation to maternal age^5^. In another study, the aneuploidy rate was 79% in morphologically high-quality embryos from subjects with a maternal age above 41, compared to 56% in subjects with a maternal age below 35^6^. Additionally, basic science research has uncovered mechanisms underlying the increased rate of aneuploidy in embryos with increased maternal age. For example, the chromosomal cohesion of a germinal vesicle (GV) oocyte progressively weakens as maternal age increases, resulting in meiotic aneuploidy^7–9^. However, there is no significant correlation between maternal age and mitotic aneuploidy during post-fertilized cell division^5^. In addition, there is no correlation between paternal age and chromosomal aneuploidy. Thus, the mother’s age and the chromosomal aberrations at the time of oocyte meiosis are strongly correlated and considered an important challenge. However, detailed molecular mechanisms underlying why the proportion of eggs displaying chromosomal aberrations rapidly increases after age 35 are not yet clear. Furthermore, the implantation rate remains between 50 and 80%, even when a euploid blastocyst is transferred^10,11^. Thus, there must be important factors that influence embryonic implantation in addition to the presence of chromosomal aberrations, such as those associated with pre-implantation embryonic development, the endometrium and embryo-endometrium interactions.

Therefore, it is important to elucidate the mechanisms underlying the regulation of the expression of genes involved in critical functions, such as meiosis, preimplantation development and implantation. It will thus be necessary to generate a standard gene expression profile of human preimplantation embryos. Several recent RNA-seq studies using samples ranging from unfertilized human eggs to preimplantation embryos have been reported^1,2^. These are valuable as standard human gene expression profiles in the early stages of development and have provided several important findings.

In mice, data can be obtained under strictly controlled experimental conditions using genetically homogeneous inbred animals. However, for humans, there is a heterogeneous genetic background, and there are subtle differences in the techniques utilized for oocyte collection, *in vitro* fertilization and embryo culture protocols at various assisted reproduction medical facilities.

To clarify the variations in the gene expression profiles of individual human embryos, we performed RNA-seq analysis of individual embryos, acquired the gene expression profiles, and analysed the correlation between parental age and gene expression.

## Results

### The overall gene expression profiles and parental age

We first evaluated the impact of parental age on the gene expression profiles of human blastocysts. Principal component analysis (PCA) was performed, and Spearman’s correlation analysis between each principal component (PC) and maternal or paternal age was calculated. The age of the donor couple and the number of embryos provided are summarized in Supplementary Table 1.

**Figure 1:**
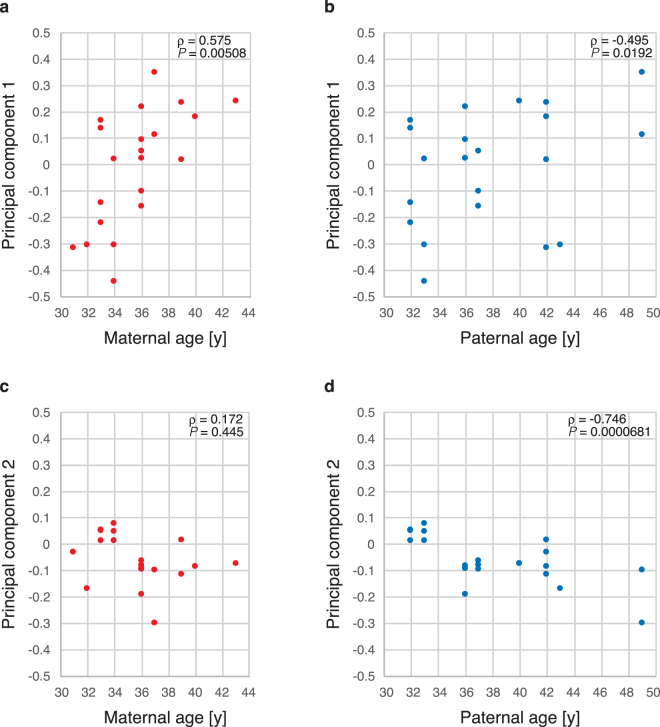
Parental age and gene expression profiles. PCs of the human blastocyst gene expression data were plotted against the maternal or paternal age. (**a**) The first PC and maternal age, Spearman’s ρ = 0.575, *P* = 0.00508. (**b**) The first PC and paternal age, Spearman’s ρ = 0.172, *P* = 0.445. (**c**) The second PC and maternal age, Spearman’s ρ = −0.495, *P* = 0.0192. (**d**) The second PC and paternal age, Spearman’s ρ = −0.746, *P* = 0.0000681.

Maternal age was significantly correlated with the first PC (Fig. 1a), but not with paternal age (Fig. 1b). The second PC was weakly correlated with maternal age (Fig. 1c) and strongly correlated with paternal age (Fig. 1d). However, it was difficult to conclude that paternal age directly affected the gene expression profile, because among the nine couples that were invited to participate in this study, paternal and maternal age demonstrated only a weak correlation (ρ = 0.554, *P* = 0.00749).

### Altered expression of genes in association with parental age

We identified genes which had significantly altered expression between the young (<35 years) and elderly mother (>35 years) groups. The young mother group included 3 couples and 11 embryos; the older mother group included 5 couples and 11 embryos. 818 and 576 genes were identified as decreased and increased, respectively (Supplementary Table 2).

The gene set displaying maternal age-dependent down-regulation was then analysed via the Database for Annotation, Visualization and Integrated Discovery (DAVID)^12^ to identify any associations with GO terms for biological functional annotation. Genes associated with the terms “nucleosome assembly”, “chromatin assembly”, “protein-DNA complex assembly”, “nucleosome organization”, “chromosome organization” and “homologous chromosome segregation” were significantly enriched in the gene set exhibiting maternal age-dependent down-regulation. The Benjamini-corrected *P* value for “chromosome organization” was 0.00145 (Supplementary Table 3).

**Figure 2:**
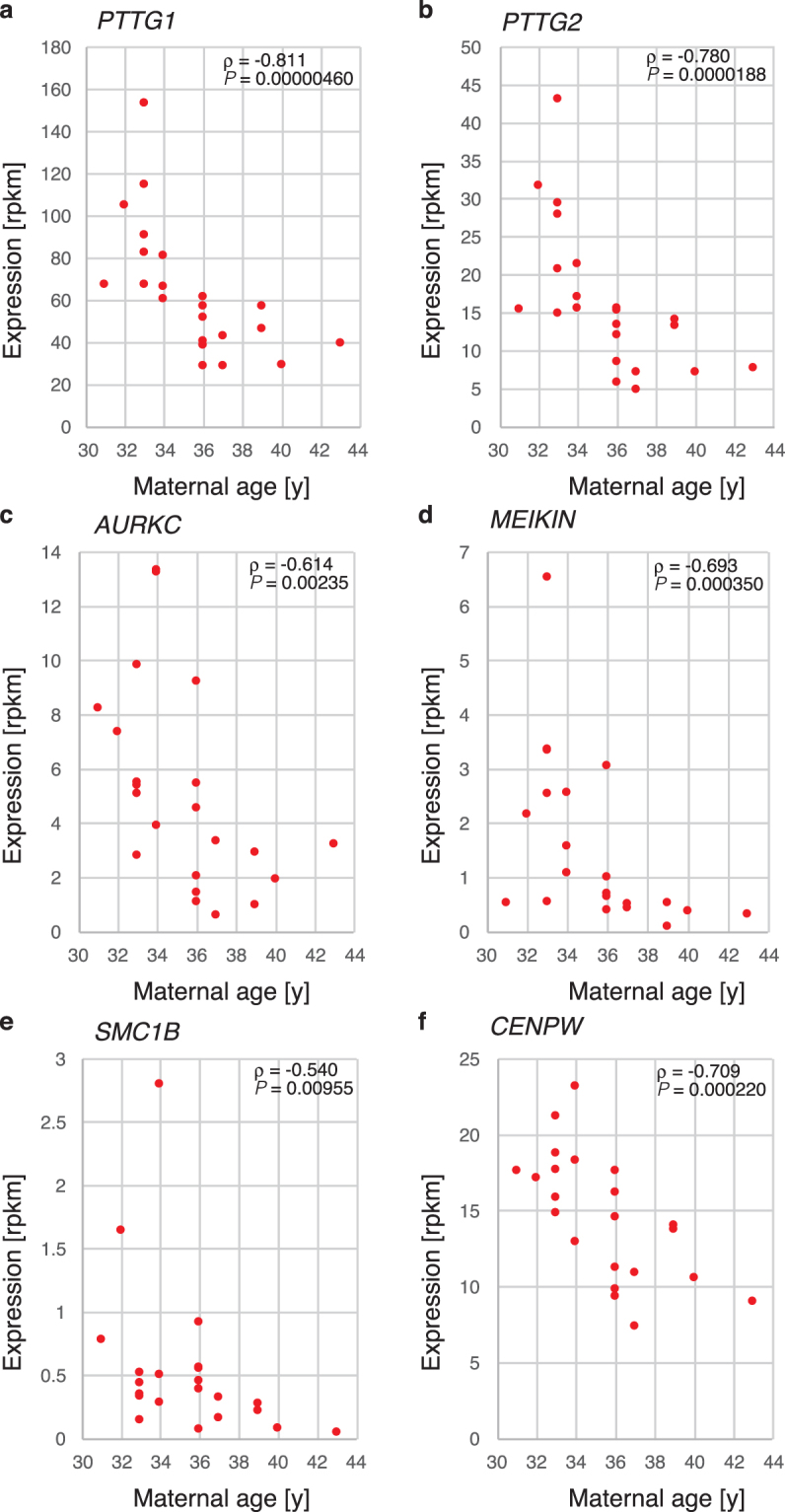
Genes that were down-regulated with advanced maternal age. The expression levels (in rpkm) were plotted against the maternal age for *PTTG1* (**a**), *PTTG2* (**b**), *AUKRC* (**c**), *MEIKIN* (**d**), *SMC1B* (**e**) and *CENPW* (**f**). The correlation coefficient ρ and *P* value between the expression and ages are also presented in each panel.

The genes associated with these terms included histones, pituitary tumour-transforming genes (*PTTGs*), a meiosis-specific kinetochore protein (*MEIKIN)* and *Centromere Protein W* (*CENPW)*. The proteins derived from these genes were important for chromosomal segregation during metaphase in the cell cycle. In particular, *PTTG1/Securin* (Spearman’s ρ = −0.811, *P* = 0.00000460) and *PTTG2* (ρ = −0.780, *P* = 0.0000188) gene expression showed the strongest negative correlations with maternal age (Fig. 2a and b). In addition, these genes demonstrated weak negative correlations with paternal age. *Aurora kinase C* (*AUKRC*) (Fig. 2c) exhibited a weaker negative correlation with maternal age (ρ = −0.614, *P* = 0.00235) than *PTTG1*.

*MEIKIN*, a meiosis-specific kinetochore protein, *SMC1B*, a subunit of the cohesin complex during meiosis, and *CENPW*, one of the inner kinetochore proteins that is required for normal chromosome organization and normal progression through mitosis, also showed negative correlations with advanced maternal age (Fig. 2d–f). As shown in Fig. 2, the gene expression patterns for *PTTG1*, *PTTG2* and *CENPW* exhibited similar distribution patterns in the blastocysts. These results suggest the existence of a common gene expression regulatory mechanism in oocytes.

**Figure 3:**
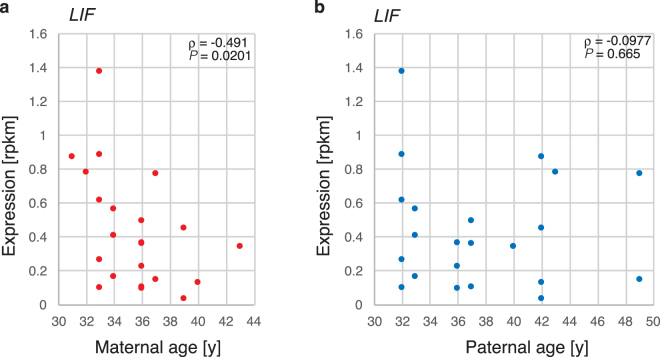
*LIF* expression according to parental age. *LIF* expression levels were plotted against the maternal age (**a**) or paternal age (**b**).

Several genes that exhibited reduced expression and were associated with advanced maternal age did not demonstrate GO term enrichment. Among these were genes that are important for preimplantation embryonic development and implantation. For example, *LIF*, which is involved in implantation, was down-regulated and found to be associated with advanced maternal age (ρ = −0.492, *P* = 0.0201) (Fig. 3a) and weakly associated with paternal age (ρ = −0.0977, *P* = 0.665) (Fig. 3b). To validate the expression levels of several genes estimated by RNA-seq, we attempted to quantify these levels from trace samples by droplet digital PCR. *PTTG1*, *AURKC* and *CENPW*, which could be robustly amplified by PCR, showed high correlation coefficients, 0.918, 0.880, and 0.791, respectively, indicating that the quantitative accuracy in our analysis was sufficient (Supplementary Figure 1). The expression levels of these genes were all significantly correlated with maternal age. Therefore, not only did they show high expression levels in the young mother group, but their expression levels also tended to decrease with maternal age.

**Figure 4:**
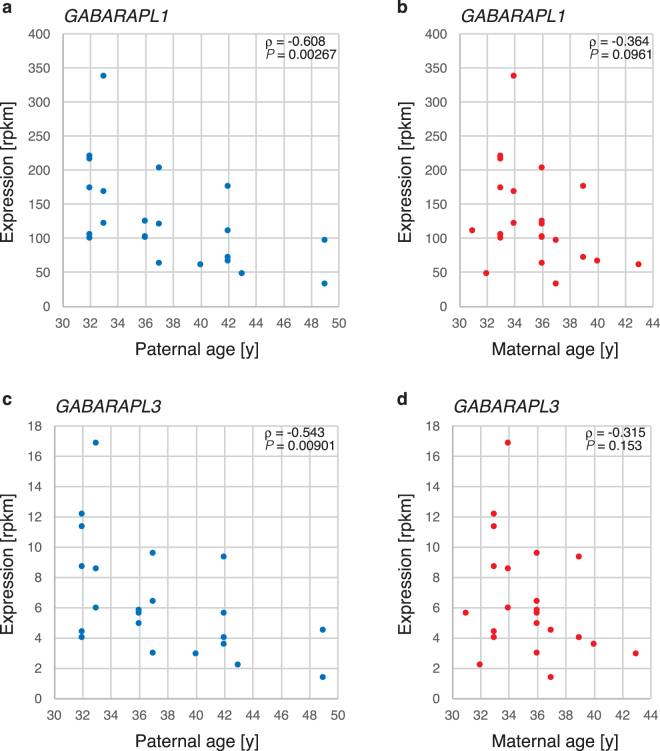
*GABARAPL1* and *GABARAPL2* expression according to parental age. *GABARAPL1* expression levels were plotted against the maternal age (**a**) or paternal age (**b**), and *GABARAPL3* expression levels were plotted against the maternal age (**c**) or paternal age (**d**).

In contrast, two genes that are important for autophagy, specifically *GABARAPL1* (ρ = −0.608, *P* = 0.00267) and *GABARAPL3* (ρ = −0.543, *P* = 0.00901), were negatively correlated with advanced paternal age (Fig. 4). However, the possibility of confounding due to maternal age could not be definitively excluded.

The expression levels of many genes change significantly as development progresses. If the development of the embryo is delayed or accelerated as the maternal age advances, changes in the expression of these genes could be explainable by a delay or acceleration of the developmental process. Using previously reported RNA-seq data^2^, we plotted the fold change in gene expression between morulae and blastocysts and the correlation coefficients of gene expression with maternal age in our study. There was no correlation found between them (Supplementary Figure 2). Therefore, embryogenesis was not delayed or prematurely accelerated as maternal age increased.

### Transcripts from repetitive sequences in which expression was correlated with maternal age

**Figure 5:**
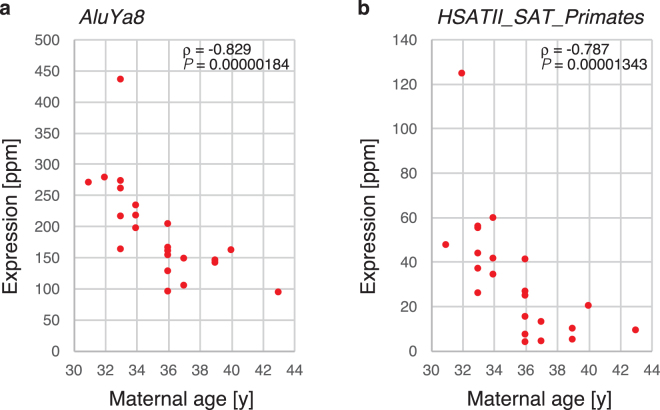
*Alu* and major satellite transcript expression according to parental age. *AluYa8* expression levels (**a**) and *HSATII* (**b**) were plotted against the maternal age.

Next, to determine whether there were epigenetic changes in the embryonic genome due to advanced maternal age, we mapped the RNA-seq data to Repbase. AluY, AluS and major satellite repeats (Fig. 5) were found at the top of the table (Supplementary Table 4). AluY and AluS are actively expressed and have only been recently incorporated into the human genome, evolutionarily speaking. However, the Alu sequence is transcribed by RNA polymerase III and does not have a poly(A) tail. In our RNA-seq analysis, reverse transcription was performed by oligo-dT. Therefore, transcripts lacking a poly(A) tail were not analysed. In fact, there were several genes that were found to have AluY sequences in the 3′ non-coding sequence. Therefore, it is possible that several of these genes did exhibit decreased expression with maternal age. Conversely, a major satellite sequence did not exist in the gene, but was concentrated in the centromere. It has been reported that the major satellite sequence is demethylated and transcribed in the germ-cell lineage^13^. Changes in the expression level of the major satellite suggest that the epigenetic state changes with maternal age.

## Discussion

Based on the PCA results for blastocyst gene expression in this study, maternal age had a strong impact on changes in gene expression profile. We identified more than 800 genes in which expression was reduced with maternal age. Thus, maternal age appears to be the strongest factor affecting the regulation of gene expression in human blastocysts. Among these genes, several interesting ones were particularly enriched. Genes associated with GO terms such as “cell cycle control” and “metaphase checkpoint regulation” that also play an important role in chromosomal segregation were over-represented among parental age-dependent down-regulated genes. PTTG1, also known as Securin, is an M-phase checkpoint protein that prevents premature sister chromatid separation and the occurrence of chromosomal abnormalities during mitosis and meiosis. Both male and female *Pttg1*-null mice are known to be subfertile^14^. Aurora kinase C (AURKC) is a kinase component of the chromosome passenger complex, which functions during meiosis to ensure correct binding between chromosomes and microtubules. In murine studies investigating *Aurkc*, a high incidence of egg and chromosomal abnormalities was observed when this function was lost^15^. MEIKIN, a kinetochore regulator that is specific to meiosis, is essential for chromosome segregation during meiosis I, and both male and female *MEIKIN*-knockout mice are infertile^16^. SMC1B is a subunit of the cohesin complex that functions specifically in meiosis, and reduced levels of this protein may cause aneuploidy during meiosis I. Chromosomal abnormalities during meiosis are frequently observed in *Smc1b*-knockout mice of advanced age^17^. However, according to one study, adequate expression of *Smc1b* during prophase I prior to the primordial follicle stage is essential and does not require subsequent expression of *Smc1b* at the prolonged resting phase in oogenesis for proper chromosome segregation in the mouse^18^. CENPW is an important protein for kinetochore and microtubule binding. Although reduced *Cenpw* expression causes chromosomal abnormalities during mitosis^19^, the precise functions of the CENPW protein during meiosis are not yet well characterized.

*PTTG1/2* demonstrated a significant negative correlation with both maternal and paternal age (Supplementary Figure 3). According to the RNA-seq data from Xue *et al*.^1^, the *AURKC* and *PTTG1/2* mRNA levels declined during development from the oocyte to the blastocyst stage (Supplementary Figure 4). Therefore, the maternal age-dependent reductions in gene expression observed in the blastocysts had likely already occurred at the oocyte stage. If gene expression levels must be maintained throughout the GV phase, reduced expression with advanced age may cause chromosomal abnormalities during the course of meiotic cell division.

According to McCoy *et al*., the absolute number of meiotic errors increases with increased maternal age, but the mitotic error rate does not change^5^. The genes that were affected by maternal age in the present study potentially cause aneuploidy during meiosis in aged women. To address this possibility in the future, we will perform blastocyst gene expression analysis via an analysis of chromosomal abnormalities or transcriptome analysis of unfertilized metaphase II (MII) oocytes. Furthermore, it will be important to identify upstream gene expression regulators associated with changes in expression that occur with advanced maternal age.

We also identified certain genes that were not associated with chromosomal abnormalities, despite reductions in their expression associated with advanced parental age, that were potentially related to embryonic growth and/or implantation. *LIF* demonstrated a significant reverse correlation with maternal age. This cytokine, which performs many biological functions, plays an important role in implantation in mammals, including humans. *LIF* is expressed in both the pre-implantation embryo itself and maternal endometrial cells^20^. Additionally, implantation disorders have been observed in *Lif* knockout mice^21^. However, although *LIF* expression in the endometrium appears to be essential for implantation in mice, its expression in the embryo itself is not necessary. In humans, *LIF* expression is observed in the endometrium of fertile women during the proliferative and secretory phases of the menstrual cycle, whereas expression is low in infertile patients who have experienced repeated implantation failures^22^. Furthermore, the levels of LIF and GP130 in the uterine luminal fluid are lower in infertile women than in fertile women^23^. Taken together, these observations suggest that *LIF* expression in the endometrium is essential for embryonic implantation. In the current study, we observed reduced *LIF* expression in human blastocysts of advanced maternal age, but this reduced expression was unlikely to be a direct cause of the low implantation rate associated with increased maternal age. Indeed, among the more than 800 genes in which expression was reduced with increased maternal age, reduced expression levels of growth factors or cytokines in the pre-implantation embryo may result in the reduced embryonic implantation rate associated with advanced maternal age.

The autophagy-lysosomal system is thought to be essential in early-stage murine embryos^24^. Autophagy in the murine embryo decreases with advanced maternal age, and embryos exhibiting reduced autophagy activity reach the blastocyst stage at a lower rate^25^. Additionally, *GABARAPL1* expression is reduced in aged porcine oocytes, but this expression is rescued with treatment with rapamycin (an mTOR inhibitor), which improves embryonic development^26^. We observed a parental age-dependent reduction in *GABARPL1* and *GABARPL3* expression. In terms of *GABARPL1*/3 expression, paternal age demonstrated a more intense reverse correlation than maternal age in our study. According to Xue *et al*.^1^, *GABARPL1* and *GABARPL3* expression is detectable at the 8-cell stage. Thus, the zygotic expression of *GABARPL1* and *GABARPL3* may contribute to pre-implantation growth through the autophagy system. The reduction in *GABARPL1* and *GABARPL3* expression in association with parental age may contribute to the lower developmental potential of embryos.

Mapping of RNA-seq data to repetitive sequences revealed that the expression of major satellite repeats increased with maternal age. We also mapped previously reported RNA-seq data of the human pre-implantation embryo^2^ against repetitive sequences, and we found that the transcripts from major satellites were highly expressed in growing oocytes. Subsequently, the expression dramatically decreased in MII oocytes; this was restored after fertilization (Supplementary Figure 5). Therefore, it appears probable that the major satellite transcripts we observed in the blastocyst stage were zygotic. It has been reported that the major satellite is highly methylated and forms heterochromatin in somatic cells. However, accumulation of hydroxymethyl cytosine in the pericentric region has been observed in germ cells, and transcripts from the major satellite are observed as demethylation progresses. This is observed in primordial germ cells (PGCs) and remains until the MII oocyte stage in females, whereas in males, hydroxymethyl cytosine can’t be recognized in the spermatocytes and spermatids^13^. In addition, transcripts derived from the major satellite are also observed after fertilization. We observed that the expression level of transcripts derived from the major satellite decreased with increasing maternal age in blastocysts. This suggests that the levels of cytosine modification in the major satellite of oocytes may also change with maternal age and be transmitted to the next generation after fertilization. It has not been determined whether cytosine modification of the centromeric major satellite repeats might affect kinetochore formation or function during meiosis. This phenomenon is also interesting in relation to the observation that chromosomal nondisjunction at meiosis increases with maternal age.

Previously, Kirkegaard *et al*. performed gene expression analysis of TE cells in implanted and non-implanted embryos, obtained via TE biopsy^27^, and identified 36 genes exhibiting reduced expression levels in the non-implanted embryos. Among these genes, six (*GUSBP2*, *LOC253573*, *TECTA*, *ATF6*, *EFNB1* and *RPS21*) were also reduced in a maternal age-dependent manner in our study. The identification of these overlapping genes suggests that the parental age-dependent genes identified in our study include certain important genes that impact embryonic implantation efficiency.

Hamatani *et al*. and Steuerwald *et al*. analysed the relationship between maternal age and the gene expression profile of unfertilized eggs of mice and humans, respectively. Certain genes involved in “cell cycle control” and “checkpoint regulation” were down-regulated with increased maternal age in murine^28^ and human oocytes^29^. In these papers, it has been reported that the expression of genes related to oxidative stress, such as thioredoxin family genes, in oocytes decreases with increasing maternal age. There was no overlap between the age-dependent genes we identified in blastocysts and Steuerwald’s data derived from older oocytes. Superficially, this appears to be a discrepancy. However, it is possible that older oocytes of low quality may not develop into blastocysts, and our blastocyst samples may have exhibited “good oocyte” bias, whereas Steuerwald’s samples did not exhibit any such bias.

To the best of our knowledge, this is the first transcriptome analysis utilizing single-embryo RNA-seq analysis of human blastocysts derived from ICSI fertilization including samples from elderly women. Based on the results of this study, parental age strongly affects the embryonic transcriptome. We identified many genes in which the expression levels were reduced with increasing maternal age. Among these, several were important for chromosomal segregation at meiosis, and others were critical for embryo growth and implantation. In addition, many genes whose relationship with embryogenesis is unknown showed similar expression changes. These included histones, transcription factors, and zinc finger proteins. Identifying the master regulator of gene expression regulation induced by parental age among these genes will be an important focus of future studies. Our gene expression studies improve our understanding of the molecular mechanisms underlying the meiotic error process, embryonic implantation and epigenetic changes induced by the parental physiological condition, including age. Greater understanding of parental age-dependent gene expression changes will allow us to develop new strategies to improve pregnancy rates in women.

## Methods

### Ethical approval

This study was approved by the Institutional Review Board of Medical Research, Tokyo Medical and Dental University, in 2013 (IRB reference number: 2011-27-3-3); the Faculty of Medicine, Tokyo Medical and Dental University, in 2012 (IRB reference number: 138); and Sanno Hospital in 2011 (IRB reference number: 12–77).

The embryos used in this research were surplus embryos from patients in assisted reproduction programmes at the Sugiyama Clinic, Tokyo, Japan; Denentoshi Ladies Clinic, Kanagawa, Japan; Kiba Park Clinic, Tokyo, Japan; Sanno Hospital, Tokyo, Japan; and Tokyo Medical and Dental University, Tokyo, Japan.

Nine couples were invited to participate in this study. Written informed consent was provided by the participants, and 22 intra-cytoplasmic sperm injection (ICSI) blastocysts were analysed. All methods, including sample preparation and RNA-seq analysis, were performed in accordance with the relevant guidelines and regulations of Tokyo Medical and Dental University.

### RNA isolation

Total RNA was purified with a PicoPure RNA Isolation Kit (Thermo Fisher Scientific) according to the manufacturer’s instructions. Each single-freeze blastocyst sample was directly lysed with 10 μl of Extraction Buffer (XB) from the kit.

### Single-embryo RNA-sequencing (RNA-seq)

RNA-seq analysis was performed on each individual blastocyst from a cryopreserved embryo. We previously performed linear cRNA amplification using a T7 RNA polymerase to perform DNA microarray analysis of a single mouse blastocyst^30^. We used the same experimental procedure to amplify RNA from individual blastocysts to construct individual RNA-seq libraries. Half of the purified total RNA from each single blastocyst was used to construct the RNA-seq library. The library was prepared for RNA-seq via complementary RNA (cRNA) amplification using a T7-RNA polymerase and an oligo-dT primer with a T7-RNA polymerase promotor sequence, followed by cDNA synthesis and library amplification. cRNA was amplified with a TargetAmp 1-Round aRNA Amplification Kit 103 (Epicentre). Amplified RNA was purified using an RNeasy MiniElute clean up kit (Qiagen) and used to construct a library with a TruSeq RNA Sample Preparation Kit v2 (Illumina), according to the manufacturer’s instructions with slight modifications. The poly(A) plus RNA fraction concentration was omitted using oligo-dT beads from the TruSeq RNA Sample Preparation Kit, and cRNA was fragmented and primed with the “Elute, Prime, Fragment Mix” from the kit. cDNA synthesis and subsequent steps were performed according to the manufacturer’s instructions.

### Validation of RNA-seq expression data by ddPCR

Total RNA was amplified by T7 RNA polymerase and purified with a Qiagen column as in the RNA-seq library construction. Then, the cRNA was reverse-transcribed by SuperScript III RTase (Invitrogen) with random hexamers as the primer. The expression levels of *PTTG1*, *AURKC*, *CENPW* and *RPL4* (as a control) were measured by a droplet digital PCR system QX100 (BioRad) with the resulting cDNA. The sequences of the PCR primers and Taqman probes were as follows:

PTTG1-FWD;GATCCTTGACGAGGAGAGAGA

PTTG1-REV;AGGAGACTGCAACAGATTGG

PTTG1-PRB;[56-FAM]ATTCCCATG[ZEN]GTGGAGAGGGCATC[3IABkFQ]

AURKC-FWD;GTGGATTTGTGGTGCATTGG

AURKC-REV;CTTGAGGATGCGTCTGTAAGT

AURKC-PRB;[56-FAM]TGCTATGAG[ZEN]CTGCTGGTGGGATAT[3IABkFQ]

CENPW-FWD;CCGCAGCAAAGGTAATTCTAAAG

CENPW-REV;TCTTTATGATCTGTTACCACCCAA

CENPW–PRB;[56-FAM]AGAGCAGAG[ZEN]GTTAGAAGTCAAAGAACA[3IABkFQ]

RPL4-FWD;AGGCTGCTGTTGGTGTTAAG

RPL4-REV;CAGGCTTCTTCTCCTCTGTAGTA

RPL4-PRB;[5HEX]TTTCCCACC[ZEN]AGAGGCTTCTTCTGC[3IABkFQ]

### Sequence mapping and data analysis

The RNA-seq library was sequenced to generate a 36-base single end using an Illumina GAIIx with a TruSeq SBS kit v5 for GA. The resulting sequence data were mapped against the human genome (hg38) using bowtie2^31^, followed by calculation of the “reads per kilobase of exons per million mapped reads (rpkm)” value for each gene using the Bioconductor package “DEGseq”^32^. Principal component analysis (PCA) was performed with “Cluster 3.0”^33^. The identification of differentially expressed genes in the two groups was performed using the “DESeq 2” package^34^ of the Bioconductor. For the mapping of the sequence data against repeat sequences with bowtie2, fasta format Repbase version 21.03 sequence data was used for the genome index. Sequence reads for each repetitive sequence was calculated as the parts per million (ppm) of total reads using SAMtools^35^.

### Statistical analysis

All statistical analyses were performed with R version 2.15.0 (The R Foundation for Statistical Computing). A Spearman’s rank order correlation test was used to determine the correlation coefficient ρ and *P* values between gene expression and parental ages. *P* values were adjusted for false discovery rates using the Benjamini-Hochberg procedure.

### Gene ontology (GO) analysis

Functional annotation was performed based on the Database for Annotation, Visualization and Integrated Discovery (DAVID) Bioinformatics Resource 38. The GO terms shown in this study summarized all similar sub-terms into an overarching term, and Benjamani-Hochberg-adjusted *P* values are shown for each representative term.

## Electronic supplementary material

Supplementary Figures

Supplementary Table 1

Supplementary Table 2

Supplementary Table 3

Supplementary Table 4
