# Supplementary material for: Parental age and gene expression profiles in individual human blastocysts
Source: Sci Rep. 2018 Feb 5;8:2380. doi: 10.1038/s41598-018-20614-8 (PMC5799158; doi:10.1038/s41598-018-20614-8)
Supplement: Supplementary file 1 — Supplementary Figures [file 41598_2018_20614_MOESM1_ESM.pdf]

# Parental age and gene expression profiles in individual human blastocysts

Kiyotaka Kawai, Tatsuya Harada, Tomonori Ishikawa, Rikikazu Sugiyama, Toshihiro Kawamura, Atsumi Yoshida, Osamu Tsutsumi, Fumitoshi Ishino, Toshiro Kubota and Takashi Kohda.

Supplementary Figure 1

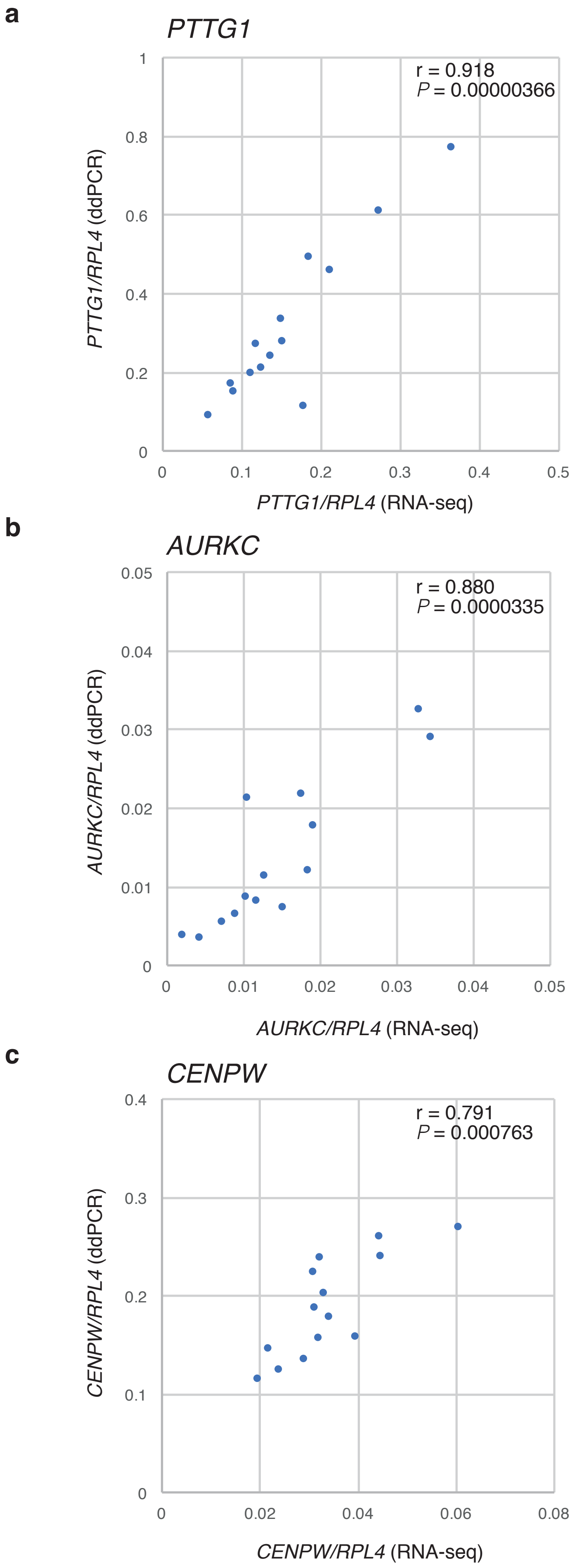


**Supplementary Figure 1.** Validation of RNA-seq expression data by ddPCR.
Normalized gene expression level for *PTTG1* (a), *AUKRC* (b) and *CENPW* (c) calculated from RT-ddPCR (y-axis) was plotted against that estimated from RNA-seq data (x-axis). Pearson' s correlation coefficients r and *P* values are also shown in each panel. *RPL4* expression level was used as a control for normalization.

Supplementary Figure 2

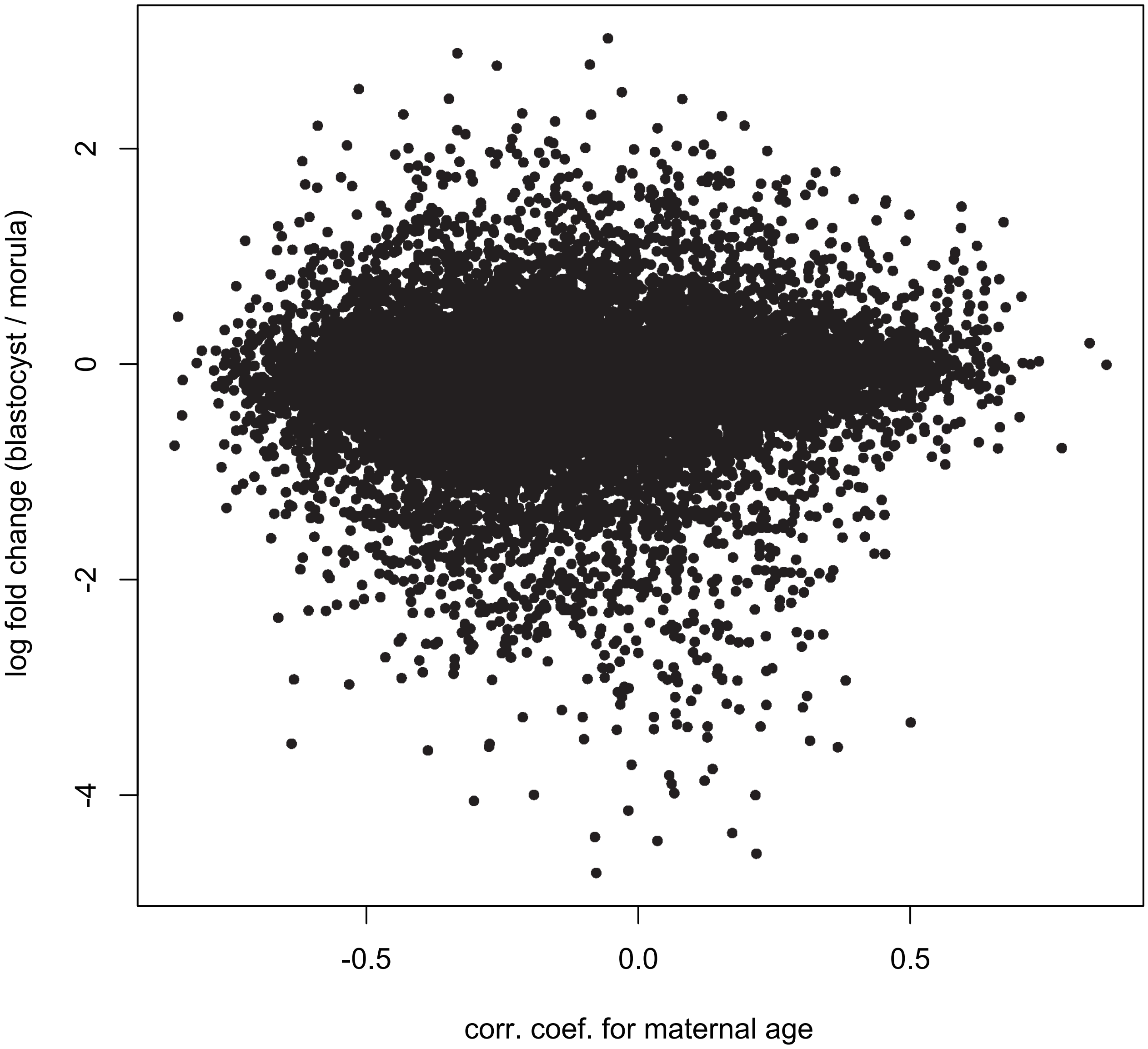


**Supplementary Figure 2.** Gene expression changes between morulae and blastocysts compared to gene expression of blastocyst-stage embryo correlation coefficients with maternal age.

The x-axis represents the correlation coefficient between each rpkm value of this study and maternal age. The y-axis represents the log fold change between the mean rpkm value of morulae and blastocysts for the data reported by Hendrickson *et al*.

Supplementary Figure 3

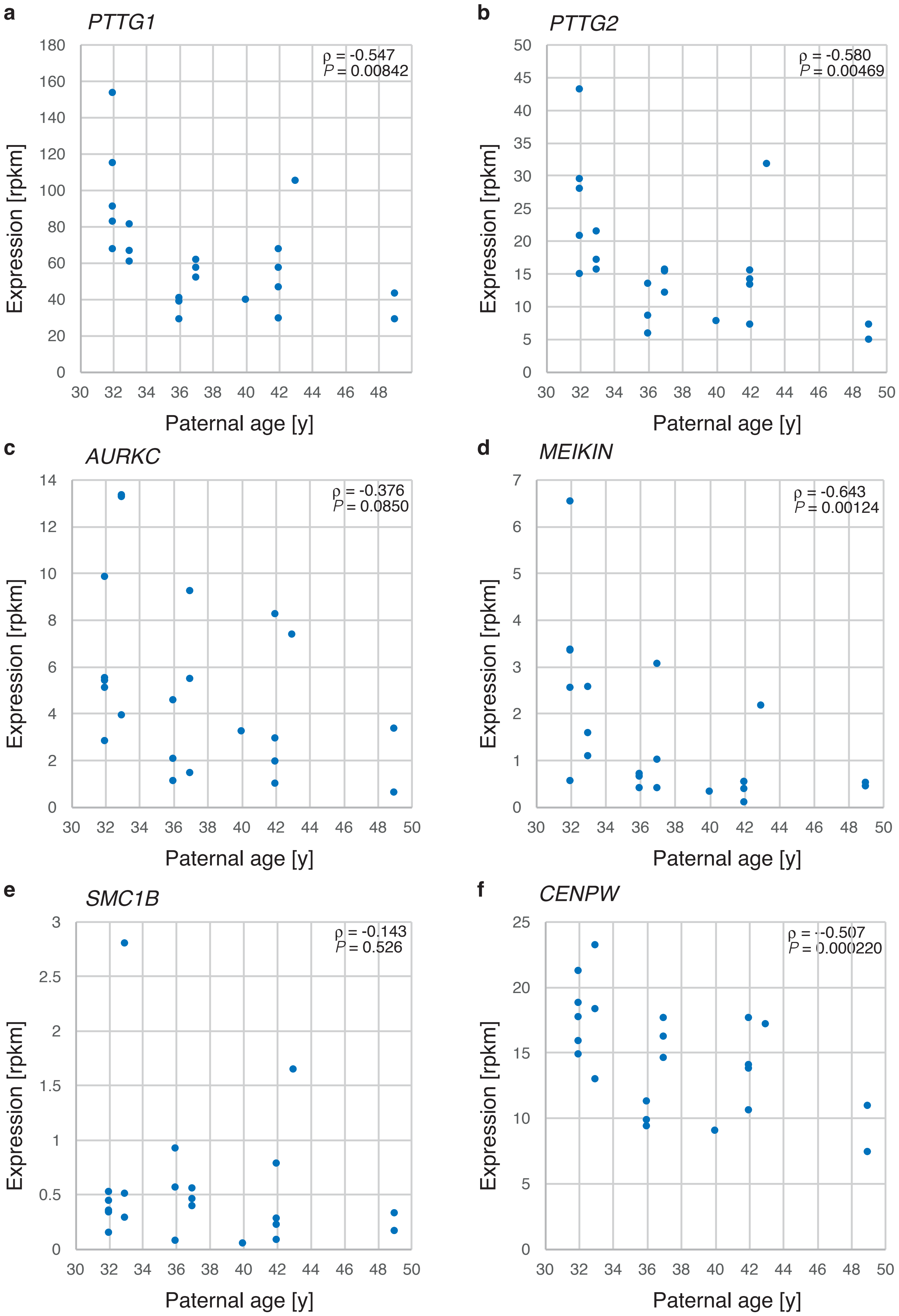


**Supplementary Figure 3.** Genes down-regulated with increased maternal age.
Expression levels in rpkm were plotted against the paternal age for *PTTG1* (a), *PTTG2* (b), *AUKRC* (c), *CENPW* (d), *MEIKIN* (e) and *SMC1B* (f). Spearman' s rank order correlation coefficients and *P* values are also shown in each panel.

Supplementary Figure 4

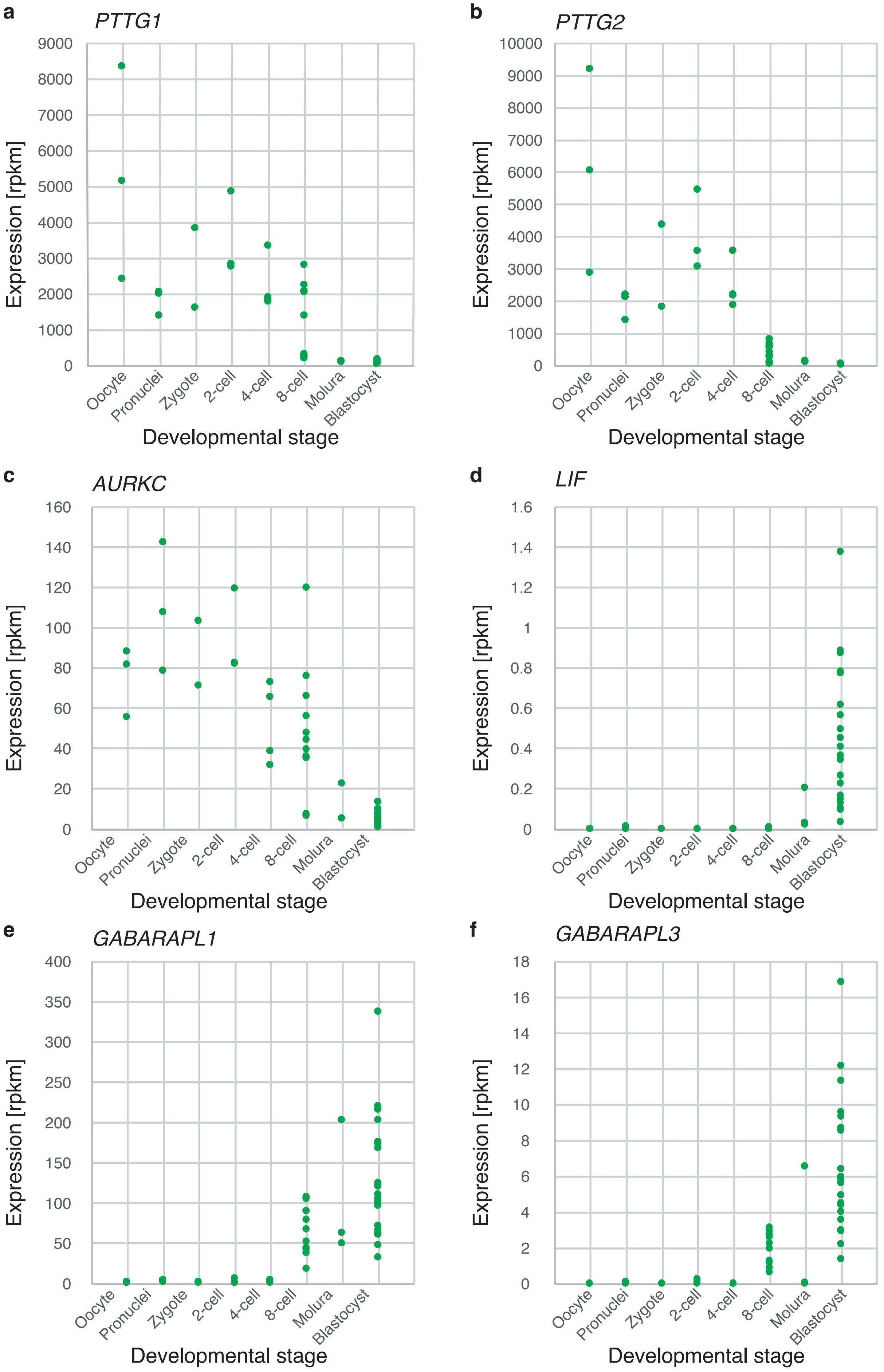


**Supplementary Figure 4.** Gene expression patterns at the pre-implantation stage for the genes down-regulated with increased parental age. Expression levels, in rpkm, were plotted during development from the oocyte to the blastocyst stage by Xue *et al*. for *PTTG1* (a), *PTTG2* (b), *AUKRC* (c), *LIF* (d), *GABARAPL1* (e) and *GABARAPL3* (f).

Supplementary Figure 5

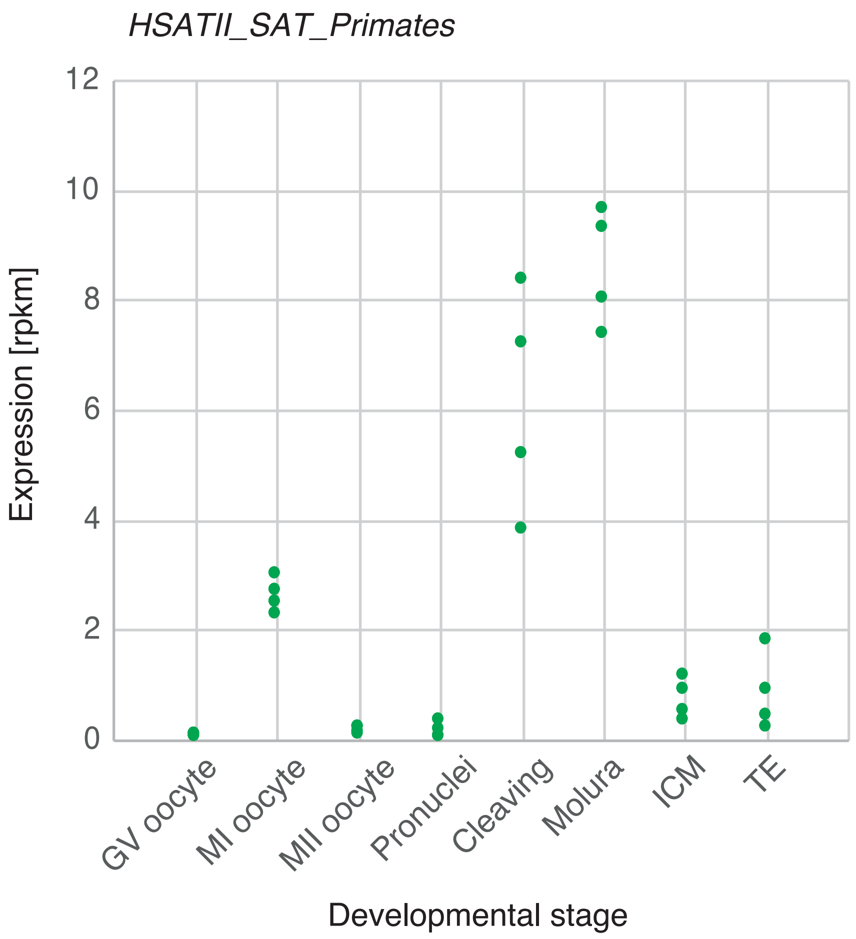


**Supplementary Figure 5.** Expression patterns at the pre-implantation stage for the transcripts from the major satellite. Expression levels in ppm were plotted during development from the oocyte to the blastocyst stage by Hendrickson *et al*. for *HSATII*.
